# The Association Between Menstrual Disorders and Workforce Participation: A Prospective Longitudinal Study

**DOI:** 10.1111/1471-0528.18109

**Published:** 2025-02-25

**Authors:** Biresaw Wassihun Alemu, Michael Waller, Leigh R. Tooth

**Affiliations:** ^1^ Australian Women and Girl’s Health Research Centre, School of Public Health, Faculty of Medicine University of Queensland Brisbane Queensland Australia

**Keywords:** employment, menorrhagia, menstrual health, morbidity, workforce participation

## Abstract

**Objective:**

To assess the association between menstrual disorders and workforce participation among Australian women.

**Design:**

Population‐based cohort study.

**Setting:**

Secondary analysis of eight surveys collected between 2000 and 2021.

**Population:**

A total of 11 152 Australian women, born between 1973 and 1978.

**Methods:**

Between 2000 and 2021, self‐reported longitudinal survey data were collected. At each survey, menstrual disorders and workforce participation were measured. Data were analysed using generalised estimating equations for multinomial responses, with stratification by age.

**Main Outcome Measures:**

Workforce participation.

**Results:**

Women who often experienced premenstrual tension reported lower odds of working part‐time compared to full‐time work (Adjusted Odds Ratio (AOR) = 0.74; 95% CI: 0.61, 0.90), with this finding strongest among women aged 31 to 40 (AOR = 0.68, 95% CI: 0.59, 0.78). While overall, women who often experienced irregular periods had higher odds of working part‐time compared to full‐time (AOR = 1.32, 95% CI: 1.08, 1.61), women aged 22 to 30 had lower odds of working part‐time (AOR = 0.61, 95% CI: 0.39, 0.97). Women who experienced severe period pain had higher odds of being unemployed compared to working full‐time (AOR = 1.18; 95% CI: 1.01, 1.36), with this association strongest in women aged 41 and older (AOR = 1.19, 95% CI: 1.01, 1.40).

**Conclusions:**

There is substantial variation in the association between menstrual disorders and workforce participation, and the role of women's ages in these associations. Increased awareness of and further elucidation of these factors may improve women's engagement in the workforce.

## 1 Introduction

Regular menstruation signals good reproductive health [1]. It is controlled by the rhythmic fluctuations of hormones [1]. Many women of reproductive age experience one or more menstrual disorders, including premenstrual tension, irregular and severe period pain and heavy menstrual bleeding [2, 3]. These disorders of menstruation are among the most frequent reasons women seek gynaecological care [4, 5].

The reported prevalence of each menstrual disorder among women varies due to diverse risk factors such as stress, age, smoking, physical exercise, contraceptive use and parity [6, 7]. For instance, period pain affects between 45% and 95% of menstruating women [8], with moderate to severe pain affecting 18%–44% of them [9, 10]. Likewise, heavy menstrual bleeding affects 25%–49% [11, 12], and irregular periods affect 5%–38%. Premenstrual tension has been estimated to affect 30%–75% of women [13, 14].

Research has shown that menstrual disorders and their symptoms can hinder women's workforce participation [15]. These symptoms can disrupt professional life, making it challenging for women to focus on tasks and maintain regular attendance, which can result in work absenteeism [15, 16]. A study in the Netherlands found that menstrual symptoms are associated with reduced work productivity [16]. Research in the USA also indicated that women with premenstrual tension report reduced work productivity and increased absenteeism [17]. Likewise, women who experienced heavy menstrual bleeding and severe period pain have reported an increased rate of work absenteeism [18, 19].

Despite this, most studies to date have been confined to cross‐sectional designs with small sample sizes, and have not adequately considered other potential covariates [19, 20, 21]. There is also a dearth of longitudinal research that has followed the same cohort of women over an extended period to examine changes in menstrual symptoms and workforce participation at different stages of a woman's reproductive life course. This is important because hormonal fluctuations and health changes, from menarche to menopause, can affect both physical well‐being and professional lives [22].

In the first few years of menstruation, many women experience irregular periods as their bodies adjust to hormonal shifts [23]. During the postpartum period, irregular periods and heavy bleeding are common and affect women's ability to work [24, 25, 26]. Additionally, caregiving responsibilities for children reduce workforce participation [27]. During menopause, women often experience more frequent irregular periods and heavier bleeding, due to oestrogen fluctuations and an increased risk of developing uterine fibroids [28]. These symptoms can further disrupt workforce participation. The Australian Longitudinal Study on Women's Health (ALSWH) offers a unique opportunity to examine these associations over 21 years of follow‐up.

## 2 Methods

### 2.1 Participants

The ALSWH is a nationwide, community‐based longitudinal study that explores various dimensions of women's health, including healthcare usage and overall well‐being, across different age groups in Australia. Since 1996, ALSWH has been collecting surveys from women in three age cohorts: those born between 1921 and 1926, who were aged 70 to 75 in 1996; those born between 1946 and 1951 (45–50 years in 1996); and those born between 1973 and 1978 (18–23 years in 1996). In 2013, a new cohort of women born between 1989 and 1995, who were 18–23 years, was recruited. The original three cohorts were randomly selected from the national health insurance database (Medicare). Women living in rural and remote areas were randomly sampled at twice the rate of women in urban areas to ensure sufficient sample size to capture the heterogeneity of health experiences of women living outside metropolitan areas. The original three cohorts have been resurveyed at approximately three‐year intervals since 1996. Further information regarding ALSWH methodology can be found in Dobson et al. [29].

**FIGURE 1:**
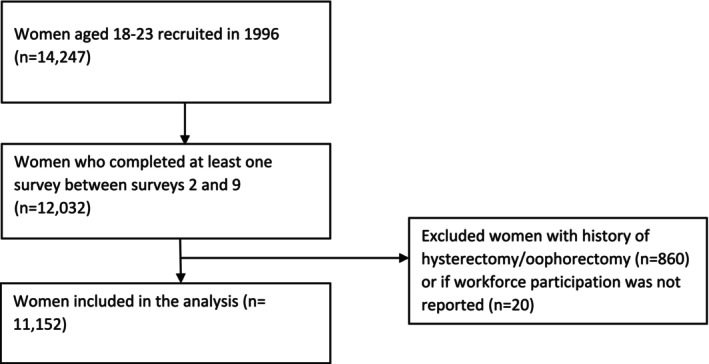
Flowchart showing the process of selecting the sample from the 1973–1978 ALSWH cohort.

For this analysis, we included women from the 1973–1978 ALSWH cohort who were aged 22–27 years at the time of their second survey in 2000 and were followed up every 3 years until survey nine in 2021. Data from survey one of the 1973–1978 cohort was not included in this analysis because most ALSWH participants at the ages of 18–23 were studying rather than participating in the workforce. Additionally, there is evidence of over‐reporting of menstrual symptoms in survey one [13]. Women who answered at least one survey between survey two and survey nine (*n* = 12 032) were considered for inclusion. Women with a history of hysterectomy or oophorectomy (*n* = 860) and those with missing outcome data (*n* = 20) were not included. The final analysis sample included 11 152 participants (Figure 1).

### 2.2 Assessment of Workforce Participation

Workforce participation was derived from the number of hours spent at work per week with the question, in a usual week, how much time, in total, do you spend doing the following things? The list included permanent part‐time work, full‐time work, casual work, and work without pay (e.g., family business). The response options ranged from 1 to 15 h, 16 to 24 h, 25 to 34 h, 35 to 40 h, 41 to 48 h, ≥ 49 h, or looking for work. The responses were categorised into three mutually exclusive groups: full‐time workers (≥ 35 h per week), part‐time workers (< 35 h per week), and unemployed (looking for work or not in labour force) [30].

### 2.3 Assessment of the Menstrual Disorder Exposures

Women were asked about their menstrual disorders at each survey using four separate questions: In the last 12 months, have you had: Premenstrual tension, irregular periods, heavy periods and severe period pain? The possible responses were measured using the 4‐category Likert scale (never, rarely, sometimes or often). Women who responded ‘often’ to any of the four questions were classified as having experienced the relevant menstrual disorder.

### 2.4 Assessments of Covariates

The study accounted for a range of covariates that were identified a priori as having potential associations with both menstrual disorders and workforce participation. These include sociodemographic characteristics such as age (in years), marital status (single, defacto or married, and divorced or widowed or separated), highest level of education (up to grade 12, trade or certificate or diploma, university degree/higher degree), and residential location (major city of Australia, inner or outer region of Australia and remote or very remote).

Health risk characteristics included Body Mass Index (BMI) classified as underweight (BMI < 18.5 kg/m^2^), normal weight (BMI 18.5–24.9 kg/m^2^), overweight (BMI 25–29.9 kg/m^2^), and obese (BMI ≥ 30 kg/m^2^) [31]. According to the guidelines of the National Health and Medical Research Council of Australia, alcohol intake was categorised as non‐drinker, low‐risk drinker, occasional drinker, risky drinker and high‐risk drinker [32]. Smoking status was classified as never smoker, ex‐smoker and current smoker. Level of physical activity was assessed using total Metabolic Equivalent (MET) values based on the frequency and duration of walking and moderate and vigorous intensity activity and categorised as nil [0–39], low [40–599], moderate [600–1199], and high [≥ 1200] MET‐minutes per week [33]. Perceived stress was measured using an 11‐item scale, with mean scores ranging from 0 to 4. Stress levels were categorised as not or somewhat stressed (0–0.9), moderately stressed (1–1.9) and very or extremely stressed (2–4) [34]. The use of contraceptives (combined oral contraceptive pill, progestogen‐only contraception, injectable, implants, vaginal ring and intrauterine device) was dichotomised into two categories (used, not used). Parity was captured by the number of live children the women had given birth to (never had children, one child, and two or more children). Pregnancy status was dichotomized into two categories (not pregnant and currently pregnant). Polycystic ovary syndrome (PCOS), endometriosis, and uterine fibroids/polyps were identified through self‐reported ‘yes’ responses to survey questions, and these conditions were tracked in subsequent surveys. Endometriosis was first assessed at survey two, while PCOS and uterine fibroids/polyps were first captured at surveys four and seven, respectively.

### 2.5 Statistical Analysis

We used multinomial logistic regression models along with generalised estimating equations (GEEs) and robust methods to assess the longitudinal association between each menstrual disorder and workforce participation [35]. The findings were presented with odds ratios (OR) and 95% confidence intervals (CIs). Full‐time workforce participation was the reference category in all models. The analyses for multinomial logistic models defined below were conducted in separate models for premenstrual tension, irregular periods, heavy periods and severe period pain. Models were adjusted for groups of time‐varying covariates: A crude model without covariates was run first (Model 1). Covariates were added in steps: Model 2 added maternal age, educational status, area of residence and marital status; Model 3 added BMI, alcohol consumption, smoking status, stress and level of physical activity; Model 4 added parity, pregnancy status, PCOS, endometriosis, uterine fibroids/polyps and contraceptive use.

ALSWH data were weighted to account for the over‐representation of women from rural and remote areas to ensure the sample reflected the broader Australian female population. However, this weighting, which was based on 1991 and 1996 Australian Census data, became misleading over time because many respondents have moved between urban, rural and remote areas since the sample was selected in 1995. Therefore, no weighting was applied in the current analysis [36].

Subgroup analyses were conducted by stratifying age into three equally spaced groups: 22–30, 31–40, and 41–50 years. These stratifications were designed to assess how the association between menstrual disorders and workforce participation differed over time and as women progressed through different lifestyle and health stages. For example, completing formal education, entering the workforce, becoming more established in careers or changing work patterns and taking on caregiver roles. As well, women may experience different patterns in menstrual disorders from their early 20s to when they may be approaching perimenopause, during which symptoms such as irregular periods, heavier bleeding, and other related health issues may become more common. R software (version 4.3.1) was used to conduct statistical analyses.

## 3 Results

### 3.1 Study Participant Characteristics

Tables 1 and 2 provide an overview of the participants' socio‐demographic details, health risk factors, reproductive information, and workforce participation. The mean (SD) age of the women at baseline was 25.2 years (± 3.1). A higher percentage of full‐time work participation was observed among women who were single, lived in major cities, had higher educational attainment, never smoked, engaged in higher levels of physical activity and maintained a healthy weight. Women who experienced lower stress levels, had no children, and were current contraceptive users also showed higher rates of full‐time work participation. A higher percentage of unemployment was observed among women living outside major cities, those who were separated, widowed or divorced, those with lower education levels, sedentary levels of physical activity, higher stress levels, two or more children and those who were obese and non‐contraceptive users. Part‐time work was more common among women who were separated, widowed or divorced, had children, did not use contraceptives, who experienced moderate stress and were underweight (Tables 1 and 2).

**TABLE 1:** Socio‐demographic, and health risk characteristics of study participants by workforce participation taken from first included survey during the follow‐up period (Survey 2–9; 89% were from Survey 2 ages 22–27).

Women's characteristics	All women (*n* = 11 152) column %	Workforce participation of women, row %			
		Unemployed (*n* = 2116)	Part‐time work (*n* = 2667)	Full‐time work (*n* = 6369)	*p* ^a^
All women	11 152 (100)	2116 (19.0)	2667 (23.9)	6369 (57.1)	
Women's age (y), mean (SD)	25.2 (3.1)	25.4 (3.2)	25.2 (3.3)	25.1 (2.9)	0.001
Area of residence, *n* (%)^b^					0.001
Major cities	5888 (52.8)	903 (15.3)	1357 (23.0)	3628 (61.7)	
Inner or outer regional	4,92 (44.1)	1126 (22.9)	1236 (25.1)	2560 (52.0)	
Remote or very remote	321 (2.9)	83 (25.9)	69 (21.5)	169 (52.6)	
Marital status, *n* (%)^b^					0.001
Single	5486 (49.2)	748 (13.6)	1303 (23.7)	3435 (62.6)	
Married or de facto	5361 (48.1)	1273 (23.7)	1282 (24.0)	2806 (52.3)	
Separated/widowed/divorced	286 (2.6)	88 (30.8)	78 (27.3)	120 (41.9)	
Educational status, *n* (%)^b^					0.001
Less than high school	1268 (11.4)	503 (39.7)	329 (25.9)	436 (34.4)	
Trade/certificate/diploma	5376 (48.2)	1149 (21.4)	1362 (25.3)	2865 (53.3)	
University degree or higher	4443 (39.8)	441 (9.9)	964 (21.7)	3038 (68.4)	
Smoking status, *n* (%)^b^					0.001
Never‐smoker	6150 (55.2)	1001 (16.3)	1436 (23.3)	3713 (60.4)	
Ex‐smoker	1826 (16.4)	461 (25.2)	454 (24.9)	911 (49.9)	
Current smoker	3163 (28.4)	650 (20.6)	773 (24.4)	1740 (55.0)	
Alcohol intake, *n* (%)^b^					0.001
Non drinker	1017 (9.1)	366 (35.9)	245 (24.1)	406 (40.0)	
Rarely drinker	3120 (27.9)	841 (27.0)	818 (26.2)	1461 (46.8)	
Low‐risk drinker	6528 (58.5)	835 (12.8)	1491 (22.8)	4202 (64.4)	
Risky drinker	473 (4.2)	72 (15.2)	111 (23.5)	290 (61.3)	
Physical activity, *n* (%)^b^					0.001
Sedentary	1056 (9.5)	308 (29.2)	228 (21.6)	520 (49.2)	
Low	3551 (31.8)	792 (22.3)	886 (25.0)	1873 (52.7)	
Moderate	2573 (23.1)	412 (16.0)	628 (24.4)	1533 (59.6)	
High	3883 (34.8)	579 (15.0)	899 (23.1)	2405 (61.9)	
Body mass index, *n* (%)^b^					0.001
Underweight	626 (5.6)	118 (18.8)	166 (26.5)	342 (54.7)	
Healthy weight	6536 (58.6)	988 (15.2)	1562 (23.9)	3986 (60.9)	
Overweight	2206 (19.8)	480 (21.8)	527 (23.9)	1199 (54.3)	
Obese	1331 (11.9)	369 (27.7)	302 (22.7)	660 (49.6)	
Stress (scores), *n* (%)^b^					
Not/somewhat stressed	6657 (50.7)	1035 (18.3)	1289 (22.8)	3333 (58.9)	0.001
Moderately stressed	4477 (40.1)	809 (18.1)	1129 (25.2)	2539 (56.7)	
Extremely stressed	976 (8.7)	254 (26.0)	238 (24.4)	484 (49.6)	

^a^ Denotes that *p*‐values were determined using Pearson chi‐square tests

^b^ Indicates the number of missing values.

**TABLE 2:** Reproductive characteristics of study participants by workforce participation taken from the first included survey during the follow‐up period (Survey 2–9; 89% were from Survey 2 ages 22–27).

Women's characteristics	All women (*n* = 11 152) column %	Workforce participation of women, row %			
		Unemployed (*n* = 2116)	Part‐time work (*n* = 2667)	Full‐time work (*n* = 6369)	*p* ^a^
Parity, *n* (%)^b^					0.001
No children	8634 (77.4)	858 (9.9)	1850 (21.4)	5926 (68.6)	
1 child	1383 (12.4)	648 (46.8)	463 (33.5)	272 (19.7)	
2 or more children	1119 (10.0)	608 (54.3)	349 (31.2)	162 (14.5)	
Contraceptive user, *n* (%)^b^					0.001
Non‐current user	5136 (46.0)	1299 (25.3)	1364 (26.6)	2473 (48.1)	
Current user	6016 (54.0)	817 (13.6)	1303 (21.7)	3896 (64.8)	
PCOS, *n* (%)^b^					0.87
Yes	488 (4.4)	82 (17.0)	117 (24.0)	289 (59.0)	
No	9228 (82.7)	1636 (18.0)	2197 (24.0)	5395 (58.0)	
Endometriosis, *n* (%)^b^					0.36
Yes	428 (3.8)	90 (21.0)	107 (25.0)	231 (54.9)	
No	10 670 (95.7)	2014 (18.9)	2547 (23.8)	6111 (57.3)	
Uterine polyps/fibroids, *n* (%)^b^					0.64
Yes	199 (1.8)	32 (16.0)	52 (26.0)	115 (58.0)	
No	7448 (66.8)	1162 (16.0)	1755 (24.0)	4531 (60.0)	
Premenstrual tension, *n* (%)^b^					0.001
Sometimes, never or rarely	9715 (87.1)	1894 (19.5)	12 292 (23.6)	5529 (56.9)	
Often	1425 (12.8)	220 (15.4)	372 (26.1)	833 (58.5)	
Irregular periods, *n* (%)^b^					0.09
Sometimes, never or rarely	10 167 (91.2)	1910 (18.8)	2420 (23.8)	5837 (57.4)	
Often	976 (8.7)	205 (21.0)	244 (25.0)	527 (54.0)	
Heavy periods, *n* (%)^b^					0.001
Sometimes, never or rarely	10 408 (93.3)	1938 (18.6)	2474 (23.7)	5996 (57.6)	
Often	736 (6.6)	176 (23.9)	191 (26.0)	369 (50.1)	
Severe period pain, *n* (%)^b^					0.95
Sometimes, never or rarely	9984 (89.5)	1892 (19.0)	2386 (23.9)	5706 (57.1)	
Often	1159 (10.4)	222 (19.1)	280 (24.2)	657 (56.7)	

*Note:* The data for *n* = 11 152 women are presented. Values are presented as both column and row percentage (%) of both Tables 1 and 2. Degree refers to an Australian University Degree.

^a^ *P* values were determined using the Pearson chi‐square test.

^b^ Missing values; area of residence, *n* = 21; marital status, *n* = 19; educational status, *n* = 65; smoking status, *n* = 13; alcohol intake, *n* = 14; physical activity, *n* = 89; body mass index, *n* = 453; premenstrual tension, *n* = 12; irregular periods, *n* = 9; heavy periods, *n* = 8; severe period pain, *n* = 9; PCOS (from survey 4‐9), *n* = 1436; stress (scores), *n* = 42; uterine polyps/fibroids, (from survey 7–9), *n* = 3505; endometriosis, (from survey 2–9), *n* = 54.

### 3.2 Menstrual Disorders

Descriptively, more women who often experienced heavy periods, compared to less often, were working part‐time or were unemployed compared to those working full‐time. On the other hand, women who often experienced premenstrual tension showed a different trend. A lower proportion of these women were unemployed, with more working part‐time and the majority being in full‐time work. There was no significant association between the severity of period pain or irregular periods and workforce participation (Table 2).

### 3.3 Association of Menstrual Disorders With Workforce Participation, With and Without Stratification by Age

The GEE analyses of the associations between each of the four menstrual disorders and workforce participation show the associations varied significantly depending on the type of menstrual symptom experienced (Table 3). In unadjusted analysis, women who often experienced premenstrual tension had higher odds of being unemployed compared to those in full‐time work (Odds Ratio [OR] = 1.06, 95% CI: 1.00, 1.13). However, this association was not significant after adjustment (Adjusted Odds Ratio [AOR] = 0.92, 95% CI: 0.82, 1.02). When stratified by age group, however, women aged 41 and older who often experienced premenstrual tension were less likely to be unemployed compared to working full‐time (AOR = 0.85, 95% CI: 0.76, 0.96) (Table 4, Figure S1). Notably, women who often experienced premenstrual tension had lower odds of being in part‐time work compared to full‐time work in both the unadjusted model (OR = 0.78, 95% CI: 0.72, 0.85) and the fully adjusted model (AOR = 0.74, 95% CI: 0.61, 0.90). The stratified analysis by age group showed this finding was strongest among women aged 31–40 (AOR = 0.68, 95% CI: 0.59, 0.78) (Table 4, Figure S1).

**TABLE 3:** Generalised estimating equation analyses of the associations between menstrual disorders and workforce participation among 11 152 women: Odds ratios (OR) and their 95% confidence interval (CI).

Variables	Unemployed versus full‐time work				Part‐time work versus full‐time work			
Premenstrual tension (Model)	Model 1, OR (95% CI)	Model 2, AOR (95% CI)	Model 3, AOR (95% CI)	Model 4, AOR (95% CI)	Model 1, OR (95% CI)	Model 2, AOR (95% CI)	Model 3, AOR (95% CI)	Model 4, AOR (95% CI)
Never/rarely/sometimes	1.00	1.00	1.00	1.00	1.00	1.00	1.00	1.00
Often	1.06 (1.00, 1.13)*	1.04 (0.98, 1.11)	1.00 (0.94, 1.07)	0.92 (0.82, 1.02)	0.78 (0.72, 0.85)*	0.77 (0.70, 0.85)*	0.76 (0.69, 0.83)*	0.74 (0.61, 0.90)*
Irregular periods (model)								
Never/rarely/sometimes	1.00	1.00	1.00	1.00	1.00	1.00	1.00	1.00
Often	1.03 (0.96, 1.10)	1.03 (0.96, 1.11)	1.00 (0.93, 1.08)	1.09 (0.96, 1.24)	1.12 (1.02, 1.23)*	1.09 (0.99, 1.19)	1.08 (0.97, 1.19)	1.32 (1.08, 1.61)*
Heavy periods (model)								
Never/rarely/sometimes	1.00	1.00	1.00	1.00	1.00	1.00	1.00	1.00
Often	0.93 (0.87, 0.99)*	0.97 (0.90, 1.04)	0.97 (0.89, 1.04)	1.05 (0.95, 1.17)	0.91 (0.83, 1.00)	0.97 (0.88, 1.07)	0.94 (0.85, 1.04)	0.91 (0.76, 1.10)
Severe period pain (model)								
Never/rarely/sometimes	1.00	1.00	1.00	1.00	1.00	1.00	1.00	1.00
Often	1.30 (1.21, 1.40)*	1.22 (1.13, 1.32)*	1.23 (1.13, 1.33)*	1.18 (1.01, 1.36)*	1.09 (0.98, 1.21)	0.96 (0.87, 1.07)	0.98 (0.87, 1.09)	1.01 (0.80, 1.27)

*Note:* Model 1: Unadjusted. Model 2: Model 1 plus area of residence, education level, marital status and age. Model 3: Model 2 plus smoking status, alcohol consumption, BMI, stress and physical activity. Model 4: Model 3 plus contraceptive use, number of children, pregnancy status, PCOS, endometriosis and uterine polyps/fibroids. 1.00, reference group.

Abbreviations: * indicates statistical significance (*p* < 0.05); AOR, adjusted odds ratio; CI, confidence interval; OR odds ratio.

**TABLE 4:** Menstrual disorders across age groups and their association with workforce participation.

Age categories	Unemployed versus full‐time, AOR (95% CI)	Part‐time versus full‐time, AOR (95% CI)
Premenstrual tension (often)		
Overall	0.92 (0.82, 1.02)	0.74 (0.61,0.90)*
22–30	0.78 (0.55, 1.09)	0.77 (0.51, 1.15)
31–40	1.05 (0.94, 1.17)	0.68 (0.59, 0.78)*
41–50	0.85 (0.76, 0.96)*	0.85 (0.69, 1.04)
Irregular periods (often)		
Overall	1.09 (0.96,1.24)	1.32 (1.08,1.61)*
22–30	0.83 (0.56, 1.21)	0.61 (0.39, 0.97)*
31–40	0.98 (0.86, 1.13)	1.09 (0.98, 1.27)
41–50	1.18 (1.03, 1.36)*	1.22 (0.96, 1.54)
Heavy periods (often)		
Overall	1.05 (0.95,1.17)	0.91 (0.76, 1.10)
22–30	0.80 (0.51, 1.27)	1.00 (0.64, 1.57)
31–40	1.12 (0.97, 1.26)	0.83 (0.72, 0.97)
41–50	1.04 (0.92, 1.17)	0.91 (0.74, 1.12)
Severe period pain (often)		
Overall	1.18 (1.01,1.36)*	1.01 (0.80, 1.27)
22–30	1.05 (0.70, 1.58)	1.18 (0.73,1.92)
31–40	1.05 (0.90, 1.22)	0.85 (0.72, 1.02)
41–50	1.19 (1.01, 1.40)*	1.11 (0.85, 1.43)

*Note:* Adjusted for area of residence, education level, marital status, smoking status, alcohol consumption, BMI, stress, physical activity, contraceptive use, number of children, pregnancy status, PCOS, endometriosis, uterine polyps/fibroids. Age was added as a covariate only in the overall result.

* Indicates statistical significance at *p* < 0.05.

Women who often experienced irregular periods had higher odds of working part‐time compared to full‐time work (unadjusted Odds Ratio [OR] = 1.12, 95% CI: 1.02, 1.23; AOR = 1.32, 95% CI: 1.08, 1.61). Of note, women aged 22–30 with irregular periods were less likely to work part‐time (AOR = 0.61, 95% CI: 0.39, 0.97). There was no overall association between women who often experienced irregular periods and being unemployed versus working full‐time (unadjusted OR = 1.03, 95% CI: 0.96, 1.10; AOR = 1.09, 95% CI: 0.96, 1.24).

Women who often experienced heavy periods had lower odds of being unemployed compared to working full‐time in unadjusted analysis (OR = 0.93, 95% CI: 0.87, 0.99). This association became null after adjustment (AOR = 1.05, 95% CI: 0.95, 1.17), and there were no associations when stratified by age.

Women who often experienced severe period pain had higher odds of being unemployed compared to working full‐time in both unadjusted (OR = 1.30, 95% CI: 1.21, 1.40) and adjusted analysis (AOR = 1.18, 95% CI: 1.01, 1.36), with this association strongest in women aged 41 and older (AOR = 1.19, 95% CI: 1.01, 1.40). There was no association between severe period pain and working part‐time versus full‐time (unadjusted OR = 1.09, 95% CI: 0.98, 1.21; AOR = 1.01, 95% CI: 0.80, 1.27) (Table 3), and no associations were found when stratified by age.

## 4 Discussion

### 4.1 Main Findings

To our knowledge, this study is the first to examine the association between menstrual disorders and women's workforce participation using a large, nationally representative longitudinal dataset collected over 21 years. By focusing on this crucial yet often overlooked aspect of women's health, our study highlights the substantial variation in workforce participation based on the type of menstrual disorders and women's ages.

The findings indicate that women who often experienced severe period pain have higher odds of being unemployed compared to working full‐time. Similarly, women who often experienced irregular periods have higher odds of working part‐time compared to full‐time. Women aged 22–30 who often experienced irregular periods were less likely to work part‐time, while those aged 41–50 were more likely to be unemployed compared to working full‐time. Conversely, women who often experienced premenstrual tension, particularly those aged 31–40, have lower odds of working part‐time compared to full‐time. These associations were independent of potential covariates, including sociodemographic, health risk and reproductive factors. We note that these associations cannot imply causality and that the retrospective nature of data collection at each survey might be prone to recall bias. However, the inclusion of multiple measures of women's reports on menstrual disorders enhances the consistency and robustness of the findings. Additionally, we addressed some of these issues in our longitudinal analyses by using time‐varying variables.

### 4.2 Strengths and Limitations

Strengths of this study were the use of a large, nationally representative sample of women and the inclusion of potential time‐varying covariates such as socio‐demographic, health risk and reproductive factors in our statistical analysis. Limitations include that we used self‐reported data to capture both menstrual disorders and women's workforce participation, which may introduce information bias. Further, the ALSWH 1973–1978 study participants were mostly from higher socio‐economic backgrounds, limiting the study's generalisability.

### 4.3 Interpretation

Our findings provide a unique contribution by highlighting the varied association of menstrual disorders and women's workforce participation and how associations differ by age. Since our study measured workforce participation by comparing part‐time, unemployment and full‐time work statuses using longitudinal data, it differs from previous studies that assessed workforce participation using other workforce outcomes, such as absenteeism and reduced productivity.

Our findings indicate that women who often experienced severe period pain have higher odds of being unemployed than working full‐time. This is consistent with other research which indicates that women suffering from period pain have significantly higher odds of being unemployed than those without the condition [37]. Some women report losing their jobs because of severe period pain [37]. The emotional and physical demands of severe period pain may contribute to women's transition out of the workforce, as untreated period pain can lead to chronic pelvic pain, further limiting women's ability to maintain employment [18, 38]. The stigma associated with menstruation in the workplace, coupled with inadequate support systems, may exacerbate these challenges [39, 40].

Our study showed that the strongest association between severe period pain and being unemployed, compared with working full‐time, was for women aged 41 and older. In contrast, cross‐sectional studies conducted by Mardon et al. [41] and Schoep et al. [16] reported that younger women have higher odds of work absenteeism compared with older women. These discrepancies may stem from the different outcomes or due to a lack of control for covariates and variations in age characteristics of the study population. Mardon et al. [41] and Schoep et al. [16] studies included women aged 15–45 years, whereas our study included women aged 22–50.

We did not observe an association between severe period pain and working in part‐time versus full‐time work, although period pain has been associated with reduced work productivity, decreased work performance and increased work absenteeism [18, 42, 43]. These discrepancies may stem from the diverse measurement techniques used to capture workforce participation across studies, as well as differences in sample characteristics. For example, Fourquet et al. [18] reported decreased work performance, while Mandeville et al. [42] reported work absenteeism due to period pain. However, the small sample sizes in these studies may limit the generalisability of their findings, and neither study adjusted for potential covariates. The use of longitudinal data and the inclusion of controlled covariates in our study could also contribute to the observed differences in findings.

Women who often experienced irregular periods were more likely to work part‐time compared to full‐time. This result is supported by a cross‐sectional study of Korean workers which revealed that women with irregular periods were more likely to work part‐time or be unemployed compared to full‐time work [44]. The unpredictable nature of irregular periods, combined with the challenges of managing other health issues that have been associated with irregular periods, such as coronary heart disease, type 2 diabetes mellitus and metabolic syndrome, may hinder the ability to secure and retain full‐time employment [45, 46]. In age‐stratified analysis, we found two noteworthy findings. Younger women aged 22–30 had lower odds of working part‐time, while older women aged 41 and older had higher odds of being unemployed compared to working full‐time. The latter finding is supported by a cross‐sectional study conducted with workers in Japan, which revealed that older women with irregular periods were more likely to report being unemployed compared to younger women [47]. Possibly, age‐related hormonal changes, such as fluctuations in oestrogen, along with conditions like uterine fibroids or endometrial hyperplasia, may contribute to menstrual irregularities and an increased likelihood of unemployment among older women [48].

We found no association between heavy periods and workforce participation, including in age‐stratified analysis. However, other evidence suggests that women experiencing heavy periods are less likely to participate in the workforce compared to those with lighter periods [21]. This discrepancy could be due to variations in the measurement of heavy menstrual bleeding, workforce participation, differences in age groups and lack of confounders. Two cohort studies have shown that heavy periods have been associated with reduced work productivity [49, 50]. However, both studies lacked control of covariates, and the investigators also acknowledged the limitation of potential sampling bias. Social norms related to workforce participation may also vary and could confound results. Similarly, differences in age groups between studies may contribute to variations in the findings.

We found that women who often experienced premenstrual tension were less likely to work part‐time compared to full‐time, with this stronger in women aged 31–40. This contrasts with another study that reported that women who experienced severe premenstrual symptoms were more likely to work part‐time compared to those with mild symptoms. However, that study could not determine whether the part‐time employment was directly due to severe premenstrual symptoms because of its cross‐sectional design, which limits the ability to establish causality. Additional research is required to validate our findings across diverse populations and age groups and to uncover the biological mechanisms.

Overall, the emotional and physical demands of menstrual disorders can place considerable strain on women, potentially leading to their transition out of the workforce. In response, some women have called for policy changes that would offer greater flexibility, such as the option to work from home or take menstrual leave during severe symptoms [51].

## 5 Conclusion

In this study, menstrual disorders were associated with women's ability to maintain full‐time work, with irregular periods increasing the likelihood of part‐time work and severe period pain associated with unemployment. In contrast, premenstrual tension may be less disruptive to full‐time work, suggesting varying levels of association with workforce participation. Notably, women aged 41 and above who experienced severe period pain and irregular periods reported higher odds of unemployment, whereas those with premenstrual tension reported lower odds of unemployment compared to full‐time work. Our findings yield insights into the association between menstrual disorders and workforce participation, emphasising the need for further research to validate these associations and elucidate underlying mechanisms.

## Author Contributions

B.W.A., M.W., and L.T. collaboratively designed the study. B.W.A. conducted the data analysis and drafted the manuscript. L.T. and M.W. critically reviewed and provided revisions to the manuscript. All authors have read and approved the final version of the manuscript.

## Ethics Statement

The ALSWH received ethical approval from the Human Research Ethics Committees at the University of Queensland and the University of Newcastle in Australia. Informed consent was secured from all participants before the completion of each survey.

## Conflicts of Interest

The authors declare no conflicts of interest.

## Supporting information


**Data S1.** Supporting Information.

## Data Availability

ALSWH survey data are owned by the Australian Government Department of Health and Aged Care, and due to the personal nature of the data collected, release by ALSWH is subject to strict contractual and ethical restrictions. De‐identified data are available to collaborating researchers where a formal request to make use of the material has been approved by the ALSWH Data Access Committee. The committee is receptive to requests for datasets required to replicate results. Information on applying for ALSWH data is available from https://alswh.org.au/for‐data‐users/applying‐for‐data/.
