# Supplementary figures and images for: The Association Between Menstrual Disorders and Workforce Participation: A Prospective Longitudinal Study
Source: BJOG. 2025 Feb 25;132(7):961–71. doi: 10.1111/1471-0528.18109 (PMC12051242; doi:10.1111/1471-0528.18109)

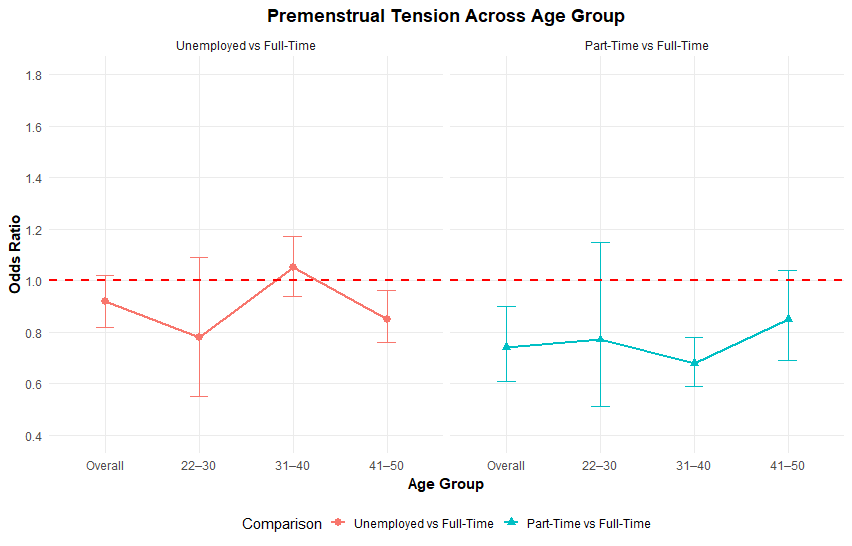


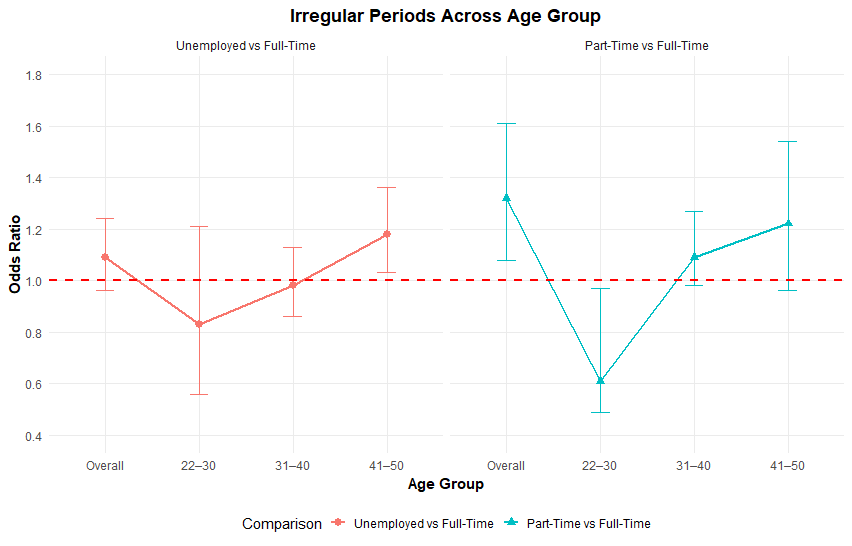


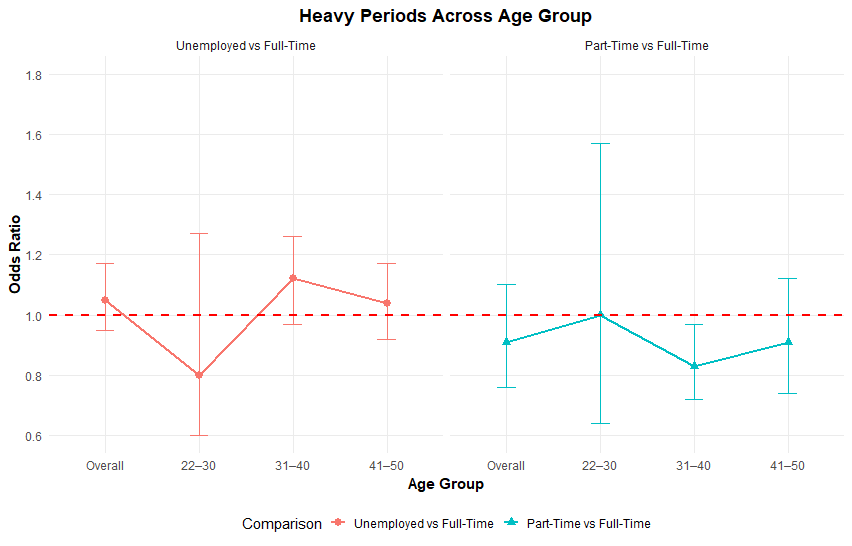


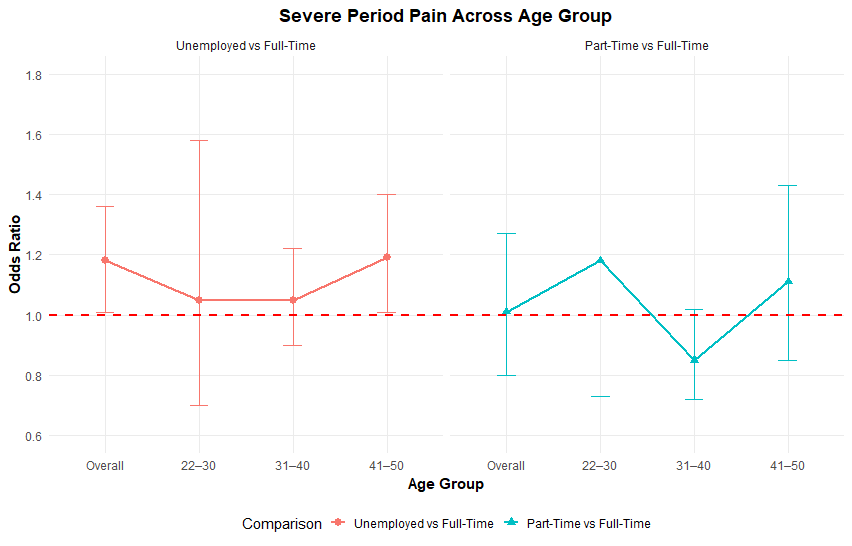

Supplement: Supplementary file 1 — Data S1. Supporting Information. [file BJO-132-961-s001.docx]
